# Electrochemical investigation of carbon paper/ZnO nanocomposite electrodes for capacitive anion capturing

**DOI:** 10.1038/s41598-022-15771-w

**Published:** 2022-07-12

**Authors:** Ebrahim Chalangar, Emma M. Björk, Håkan Pettersson

**Affiliations:** 1 Department of Science and Technology, Physics, Electronics and Mathematics, Linköping University, SE-60174 Norrköping, Sweden; 2 School of Information Technology, Halmstad University, 301 18 Halmstad, Sweden; 3 Nanostructured Materials, Department of Physics, Chemistry and Biology, Linköping University, 581 83 Linköping, Sweden; 4 Solid State Physics and NanoLund, Lund University, Box 118, 221 00 Lund, Sweden

**Keywords:** Nanowires, Synthesis and processing, Composites, Chemical physics, Electronic materials

## Abstract

In this work, we demonstrate an effective anion capturing in an aqueous medium using a highly porous carbon paper decorated with ZnO nanorods. A sol–gel technique was first employed to form a thin and compact seed layer of ZnO nanoparticles on the dense network of carbon fibers in the carbon paper. Subsequently, ZnO nanorods were successfully grown on the pre-seeded carbon papers using inexpensive chemical bath deposition. The prepared porous electrodes were electrochemically investigated for improved charge storage and stability under long-term operational conditions. The results show effective capacitive deionization with a maximum areal capacitance of 2 mF/cm^2^, an energy consumption of 50 kJ per mole of chlorine ions, and an excellent long-term stability of the fabricated C-ZnO electrodes. The experimental results are supported by COMSOL simulations. Besides the demonstrated capacitive desalination application, our results can directly be used to realize suitable electrodes for energy storage in supercapacitors.

## Introduction

Accessing freshwater, one of the most fundamental human needs, has become more and more challenging during the past decades. The continuous growth of population and energy consumption, together with alarming climate changes, is expected to have an increasingly negative impact on Earth’s living conditions and quality of life. Currently, more than one billion people around the world are suffering from a lack of clean water and its adverse consequences on health, food, and energy^1^.

A key technology that could mitigate this trend is the development of cheap, long-term stable and effective systems for combined seawater desalination and ion purification. The existing and well-developed desalination techniques, including thermal distillation and reverse osmosis, are not affordable everywhere due to high energy consumption and operational costs. Capacitive deionization (CDI) is a promising alternative technology with higher energy efficiency and lower cost^2,3^.

The CDI technology is a membrane-free, low-voltage and low-pressure operating desalination process where brackish water flows through a set of porous conductive electrodes of a capacitor under an applied external voltage. The applied potential forms a polarized electric field between the electrodes, attracting the anions to the anode (+) and cations to the cathode (−). An electrical double layer is established at the interface between the saline electrolyte and the electrode surfaces, resulting in electro-adsorption of ions on the opposite potential electrode surface. When the electrodes are saturated with captured ions, a regeneration step is carried out where the ions are desorbed by applying a reversed electrode potential^4,5^.

The performance of a CDI cell depends on the ion adsorption capacity, which is directly governed by the active surface area, wettability, surface charge density, conductivity and pore structure of the electrodes. So far, carbon-based materials, e.g., graphene^6,7^, carbon nanotubes^8^, carbon fiber textures, and carbon aerogels^9^, have proven to be the most suitable materials for CDI electrodes in terms of surface area, stability and conductivity. However, the efficiency of these materials is limited due to the hydrophobic nature of carbon materials and the active surface area. Several surface treatments, e.g., chemical modification^10^ and porosity adjustment^5^, have been proposed and studied to improve the surface properties of carbon-based materials. Alternatively, the active surface area, hydrophilicity and surface energy of the electrodes can be tuned by forming different nanostructures on their surface^11^. Decorating carbon cloths with ZnO and TiO_2_ nanoparticles (NPs), ZnO nanorods (NRs), and nickel copper hydroxide have previously shown an enhancement of the desalination efficiency^12–15^. However, those reports suffer from a low homogeneity of the grown NRs and a lack of in-depth electrochemical investigation of the final electrodes^16,17^.

In this work, we report on our development of disruptive electrodes made of a special porous carbon paper (C-paper), decorated with high-quality ZnO nanostructures, for brackish water desalination and ion purification. A highly uniform thin film of ZnO NPs was first deposited on the surface of the carbon fibers (C-fibers) in the C-paper by an inexpensive sol–gel technique. This compact layer of ZnO NPs acts as a seed layer for the subsequent growth of ZnO NRs. The electrochemical properties of the fabricated electrodes, including adsorption capacity and energy efficiency, were investigated at length and further supported by theoretical simulations. The main novelty of this study is the demonstrated low-temperature and inexpensive synthesis of high-capacity C-paper/ZnO NR electrodes with excellent uniformity which can perfectly permeate water through its porosities. The electrodes´ large open pores makes them perfectly suitable in different flow-by or flow-through CDI cell architectures^5,8^ with lower water flow resistance. The excellent performance of these electrodes points out a new route for the development of green low-cost CDI cells.

The double-layer ion adsorption mechanism in CDI is basically the same as the energy storage mechanism in supercapacitors^18^. From this perspective, our achievements in developing C-ZnO electrodes for water treatment are equally applicable for realizing supercapacitors.

## Experimental methods

All the chemicals used in this work were used as received without any further treatment or purification. Carbon papers MGL190, consisting of C-fibers with a diameter of 7 µm and a length of up to 1.5 mm, with a thickness of 190 µm, a porosity of 78%, a BET specific surface area of 0.55 m^2^/g, an electrode surface mass density of 8.4 mg/cm^2^, and an electrical resistivity of 75 mΩ.cm were purchased from AvCarb.

### Deposition of ZnO NP seed layers on C-fibers

In general, carbon-based substrates are hydrophobic and therefore inappropriate to integrate with an aqueous medium. This drawback reduces water penetration into the pores and consequently leads to a significant reduction of the active surface area of the electrodes. For the same reason, it is also problematic to uniformly grow other nanostructures on a hydrophobic surface by a solution-based growth technique. Several surface treatments, including chemical oxidation by strong acids^19,20^, introducing surface-charged functional groups^10^, using additives in the solution^15^, or applying UV-ozone treatments^21^, have been proposed to improve the surface compatibility of carbon-based materials. However, these surface modifications usually introduce numerous surface defects and undesirably changes in the surface properties of the carbon materials.

In contrast to water, some organic solvents, e.g., ethanol, can perfectly wet the C-paper substrate due to their hydrophilic nature. Therefore, ZnO NP sol–gel solutions consisting of zinc acetate (Zn(CH3COO)_2_·2H_2_O), monoethanolamine (ethanolamine), and aluminum nitrate (Al(NO_3_)_3_.9H_2_O) dissolved in 100 ml of pure ethanol were prepared with the respective final concentrations of 375 mM, 375 mM and 7.5 mM^22^. The solutions were stirred at 60 °C for 10 h and at room temperature overnight. A specified amount of aluminum nitrate was added to dope the ZnO NPs with 2 at% Al^23,24^. This degenerate doping level of ZnO transforms its electrical conductivity from that of a semiconductor to a metal.

Carbon papers were cut into 1 × 2 cm^2^ pieces and subsequentially cleaned using ultrasonication in acetone, isopropanol, and DI water, followed by drying in an oven at 110 °C for 1 h. The dried C-papers were dipped into the Al-doped ZnO sol–gel solution and pulled out at a constant speed of 30 mm/s using an automated dip-coater. The ZnO-seeded C-papers were annealed in air at 300 °C for 5 min to remove the residuals and crystallize the ZnO NPs. A further extended heat treatment would damage the C-fibers.

### Growth of ZnO NRs on seeded C-papers

ZnO NRs were grown on seeded C-papers using an inexpensive low-temperature chemical bath deposition (CBD) technique. A 100 ml equimolar aqueous growth solution of zinc nitrate (Zn(NO_3_)_2_.9H_2_O) and hexamethylenetetramine (HMT), with concentrations of 50 mM, was prepared. To simultaneously dope the ZnO NRs with Al, 2 mM aluminum nitrate (Al(NO_3_)_3_.9H_2_O) was added to the growth solution. The pH of the solution was adjusted to 6–7 by adding enough ammonia to the solution. The composition of the bath solution was chosen to obtain a final Al dopant concentration of 2 at% in the ZnO NRs^23,24^, resulting in a high electrical conductivity of the NRs. The effect of Al-doping on the crystallinity of ZnO NRs and its bonding quality to carbon-based materials has been reported in our previous work^25^.

### Electrochemical analysis

All electrochemical measurements, including cyclic voltammetry (CV) and chronopotentiometry (CP), were carried out at room temperature in a 20 mM NaCl aqueous electrolyte after three limit cycles to reach a quasi-steady behavior, using a three-electrode system. The three-electrode system comprised a 1 cm^2^ C-ZnO working electrode, kept at a distance of 1 cm from a platinum counter electrode, and an Ag/AgCl reference electrode.

## Results and discussion

**Figure 1:**
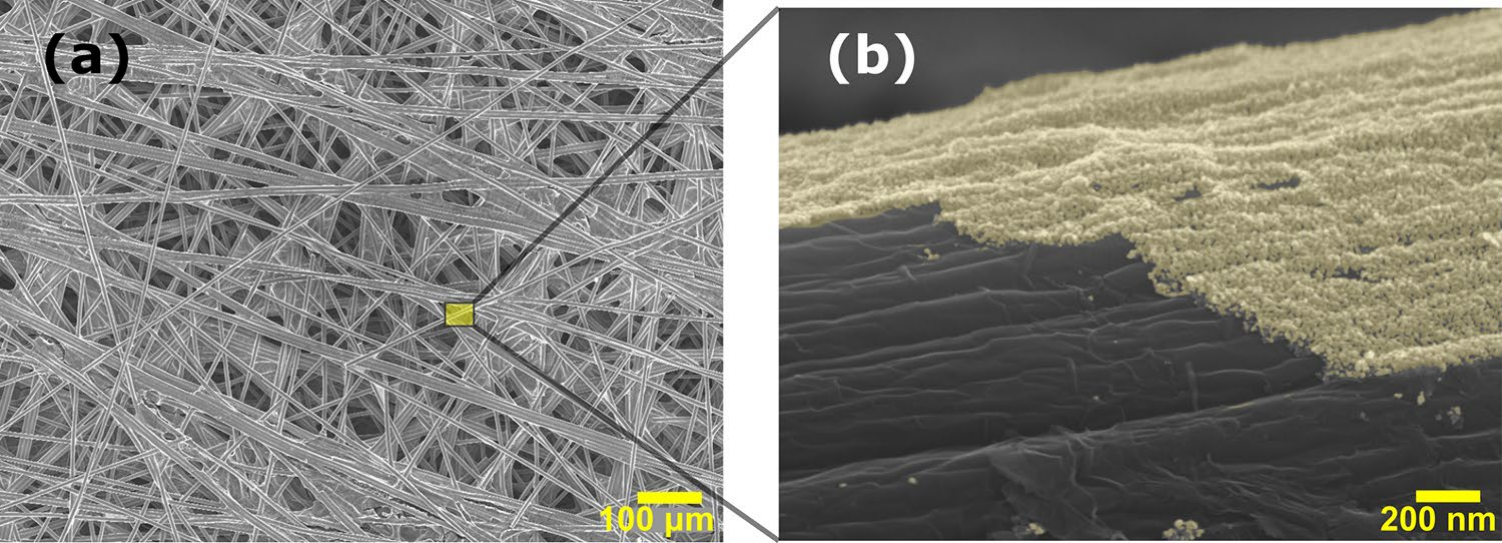
(**a**) Scanning electron microscopy (SEM) image of the C-paper coated with Al-doped ZnO NPs. (**b**) Close-up image of a thin uniform layer of Al-doped ZnO NPs (artificially colored layer) deposited on the surface of a C-fiber.

The C-papers were perfectly coated with a thin uniform layer of Al-doped ZnO NPs using the sol–gel method. Figure 1a shows the C-fiber network in a C-paper after seeding with ZnO NPs and annealing. The high-resolution SEM image in Fig. 1b reveals the resulting 30 nm thick homogeneous and dense ZnO layer on a hydrophobic C-fiber surface. Additional cross-sectional imaging reveals a full coverage of all C-fibers with a ZnO seed layer through the entire bulk of the C-paper. This seed layer is highly hydrophilic due to the high surface area of the ZnO NPs, which transforms the carbon paper from a hydrophobic substrate to a desired water-permeable porous substrate. Figure S1 demonstrates the drastically improved hydrophilicity of a C-paper after growth of ZnO NRs.

**Figure 2:**
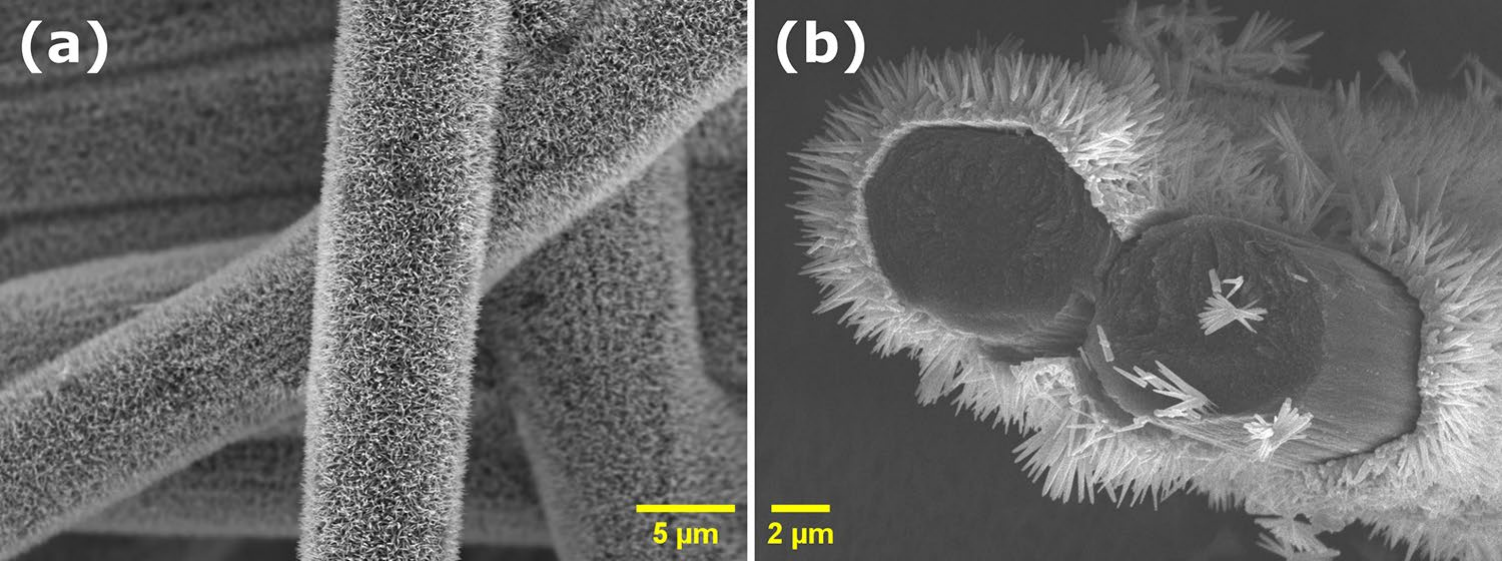
SEM images of C-fibers coated with Al-doped ZnO NPs/NRs from (**a**) a top view and (**b**) a cross-sectional view.

Subsequently, ZnO NRs with and without Al-doping were grown on the seeded C-paper by a cost-effective low-temperature CBD technique. The final nanocomposite structure is shown in Fig. 2a. The cross-sectional imaging shown in Fig. 2b reveals a C-fiber core structure coated with a compact ZnO seed layer/NR shell. The presence of NRs on the surface of the fibers dramatically increases the porosity, and thereby the available surface area, of the electrode, which is important to obtain a high desalination efficiency. The increased surface area was confirmed by Kr adsorption using an ASAP2020 (Micromeritics) which showed BET specific surface areas of 0.55, 1.29 and 2.07 m^2^/g for the pristine C-paper, C-paper with ZnO NPs and C-paper with ZnO NRs, respectively. The samples were degassed at 140 °C for 4 h prior to the measurements and the BET analysis was performed at a relative pressure range of 0.12–0.20. The isotherm adsorption plots are shown in Fig. S2.

In addition, the NRs with an average length of 2 µm and a diameter of 100 nm lead to a beneficial electric field enhancement at the surface of the electrode during the desalination when an external potential is applied^13,14^. This field enhancement was confirmed by detailed COMSOL simulations as discussed below.

**Figure 3:**
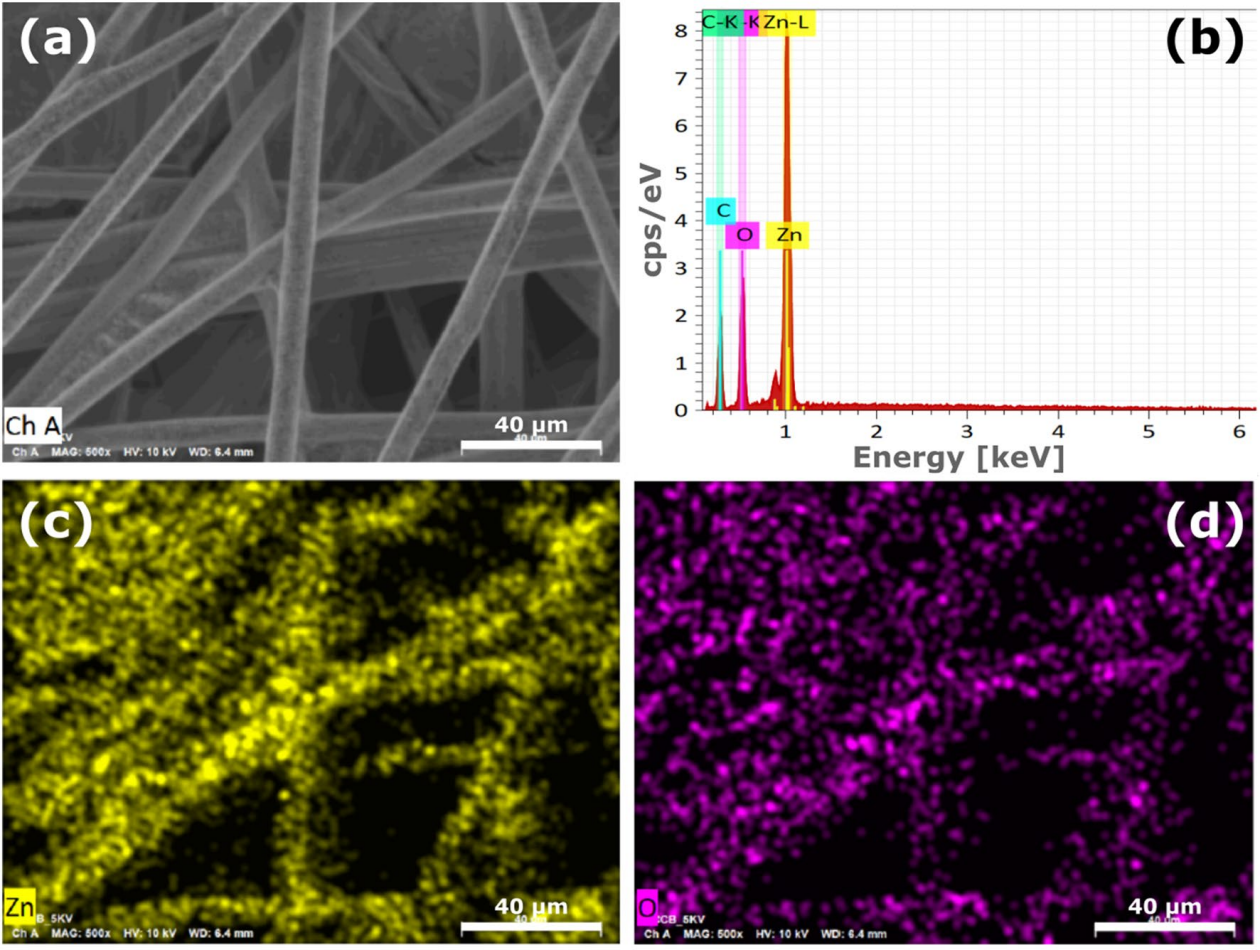
(**a**) SEM image of a typical C-ZnO:Al(NRs) sample with (**b**) corresponding EDS spectra, and spatial EDS mapping of (**c**) Zn and (**d**) O in the same area.

Figure 3 shows the spatial distributions of Zn, C and O in the sample imaged in Fig. 2, measured by energy-dispersive X-ray spectroscopy (EDS). The representative EDS spectra in Fig. 3b show the presence of C, Zn and O in the same area as shown in Fig. 3a. Figures 3c, and 3d unravel a uniform distribution of Zn and O on the surface of the coated C-fibers in Fig. 3a. The Al dopant was not identified due to its low concentration in the nanocomposite.

To investigate the ion adsorption capacity of different C/ZnO samples, four types of CDI electrodes were fabricated: (i) bare C-paper, (ii) seeded C-paper with ZnO NPs (C-ZnO(NPs)), (iii) C-ZnO(NPs) with grown ZnO NRs (C-ZnO(NRs)), and (iv) C-ZnO(NPs) with grown Al-doped ZnO NRs (C-ZnO:Al(NRs)) and assessed regarding their electrochemical properties.

**Figure 4:**
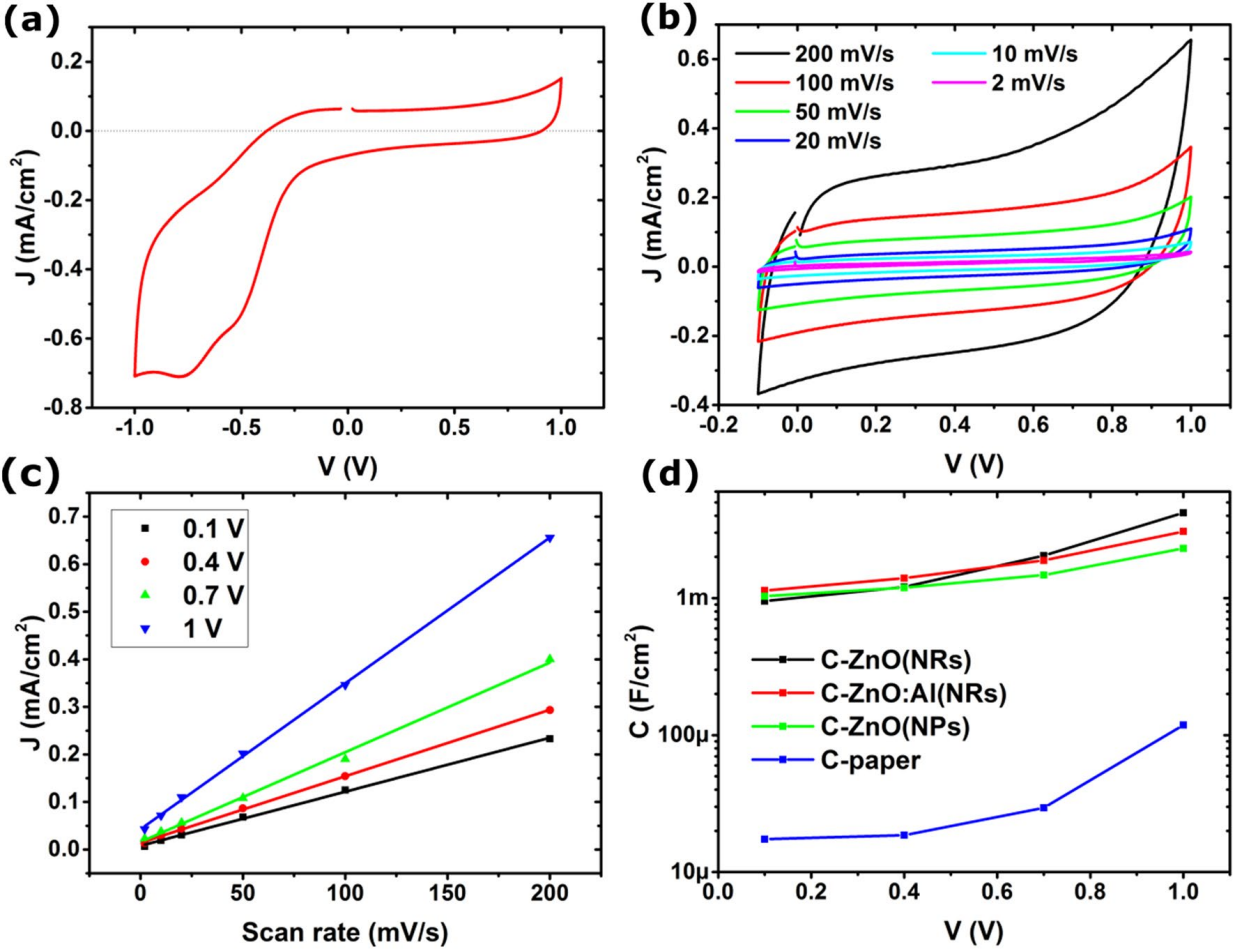
Cyclic voltammograms of a C-ZnO(NRs) electrode at (**a**) a scan rate of 50 mVs^−1^ and (**b**) different scan rates. (**c**) Current density versus scan rate, extracted from (**b**) and Fig. S3 at four different electrode potentials. (**d**) Calculated differential areal capacitance versus potential for all four investigated CDI nanocomposite electrodes immersed in the same 20 mM NaCl electrolyte.

Cyclic voltammetry characterization of a C-ZnO(NRs) electrode in a 20 mM NaCl aqueous electrolyte with the salinity of 0.12% reveals two different types of behavior in the voltage range below and above − 0.2 V (Fig. 4a). The current density varies non-linearly with the electrode potential below − 0.2 V with clear distinguishable peaks. These characteristic features indicate Faradaic reactions where a charge is transferred across the electrode/electrolyte interface^26,27^. This charge transfer is due to oxidation (electron loss) or reduction (electron gain), so-called redox reactions in the electrode immersed in the electrolyte. Applying a negative potential to our samples can irreversibly reduce ZnO to Zn atoms, resulting in the formation of undesired by-products and electrode poisoning.

In the positive potential range, an almost constant current density versus electrode potential suggests a capacitive charge accumulation in an electric double layer close to the electrode surface. In this case, no charge is transferred across the interface. Instead, the anions in the electrolyte are adsorbed onto the electrode surface due to the inner opposite charges induced by the external potential^27,28^. The capacitive (non-Faradaic) ion adsorption is usually a reversible reaction that allows regeneration of the electrodes many times without causing any damage. The presence of both Faradaic and capacitive reactions leads to a superposition of current responses, a so-called pseudocapacitive behavior of the materials^28^. Our results show that the present electrode can only operate properly in the positive potential range, and thus only act as an anode to capture anions (Cl^−^ in our electrolyte). A complete asymmetric CDI cell can be assembled by including an appropriate electrode (cathode) for cation (Na^+^) capture.

With the assumption that the current response of the electrodes, within a specific potential range, is only due to capacitive charge storage, the differential areal capacitance, C (F/cm^2^), of the electrochemical double-layer is given by the relation:

1 $${\mathrm{C} }={\frac{1}{A}}{\frac{\partial Q}{\partial V}}={\frac{J}{{{dV}}/{dt}}}={\frac{J}{\nu }} ,$$

Here Q is the charge, V is the potential, ν is the potential scan rate (V/s), J is the current density (A/cm^2^), and A is the electrode area. Equation 1 suggests that CV measurements at various scan rates (ν) can be deployed to determine the capacity of the CDI electrodes^29^. The cyclic voltammograms of a C-ZnO(NRs) electrode recorded at different scan rates shown in Fig. 4b confirm the excellent capacitive behavior of the electrode in the given voltage range. By plotting the extracted current density J from Fig. 4b versus scan rate and fitting the data to straight lines as shown in Fig. 4c, the voltage dependence of the capacitance was extracted. Figure 4d shows the differential areal capacitance of the four different investigated CDI electrodes at different biases. Assuming that the stored charge stems from Cl^−^ capturing from the electrolyte, the desalination efficiency of a complete CDI cell, including a further developed cathode, is proportional to the electrode areal capacitance^30^. The results show a significant increase in capacitance, from 30 µF/cm^2^ for the bare C-paper to 2 mF/cm^2^ at a potential of 0.7 V for the C-ZnO(NRs) electrode.

**Figure 5:**
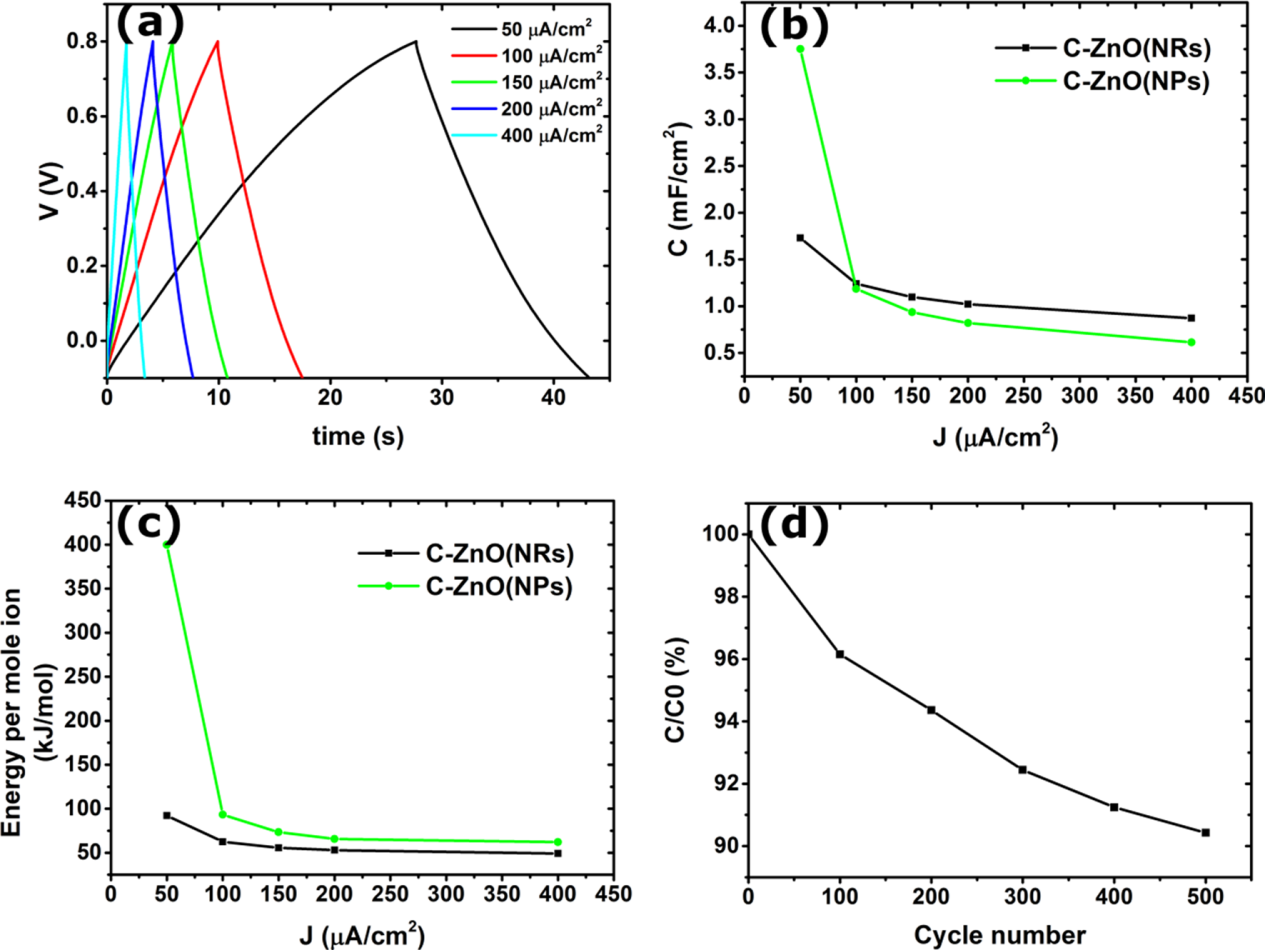
(**a**) CP curves for a C-ZnO(NRs) electrode at different charging/discharging current densities. The corresponding CP curves for a C-ZnO(NPs) electrode are shown in Fig. S4. (**b**) Integral areal capacitance versus current density for the C-ZnO(NRs) and C-ZnO(NPs) electrodes. (**c**) Calculated energy consumption per mole of Cl^−^ in a NaCl electrolyte for C-ZnO(NRs) and C-ZnO(NPs) electrodes. (**d**) The ratio of integral areal capacitance to initial capacitance for a C-ZnO(NRs) electrode after 500 cycles of charging/discharging at a current density of ± 200 μA.cm^−2^.

In addition, complementary CP, or equivalently galvanostatic charge/discharge, experiments on C-ZnO(NRs) and C-ZnO(NPs) electrodes were carried out. Figure 5a shows a conventional triangular-shaped charge/discharge cycling behavior^31^ of a C-ZnO(NRs) electrode immersed in a NaCl electrolyte. For each cycle, the electrode is first charged with a constant current to a certain voltage (0.8 V in our experiments) and then discharged with the same reverse constant current while its potential is measured. In the charging cycle, Cl^−^ are adsorbed on the electrode surface and desorbed during the discharging (regeneration) cycle. Both CV and CP results confirm the capacitive storage mechanism in C-ZnO(NRs) electrodes immersed in a NaCl electrolyte. The integral areal capacitance C_int_ (F/cm^2^) at different current densities, deduced from constant current CP measurements, is extracted using Eq. 2,

2 $${C}_{int}=\frac{1}{A}\frac{\Delta Q}{\Delta V}=\frac{J \Delta t}{\Delta V} ,$$

where J is the constant charge/discharge current density, Δt is the charging time, and ΔV is the electrode potential variation. Figure 5b shows the integral areal capacitance for both C-ZnO(NRs) and C-ZnO(NPs) electrodes. The capacitance of both electrodes is reduced at high current densities, while the C-ZnO(NRs) electrode exhibits a slightly higher capacitance compared to the ZnO-seeded carbon paper electrode.

3 $${E}_{charging}={\int }_{0}^{{Q}_{charging}}V dq=I {\int }_{0}^{{T}_{charging}}V dt$$

4 $$EPM = \frac{{E_{{charging}} }}{{{{{{\Delta }}Q_{{discharging}} } \mathord{\left/ {\vphantom {{{{\Delta }}Q_{{discharging}} } {z.q_{e} .N_{A} }}} \right. \kern-\nulldelimiterspace} {z.q_{e} .N_{A} }}}}$$

By calculating the energy supplied during each charging cycle, E_charging_, (Eq. 3), and dividing it by the number of adsorbed moles of ions during each discharging cycle, the energy consumption per mole of ions (EPM) is determined (Eq. 4)^32^. Here, ΔQ_discharging_ is the total charge desorbed during the discharging cycle, z is the charge number of the ions (= 1 in our case of Cl^−^), q_e_ is the elementary charge (1.6 × 10^–19^ C), and N_A_ is the Avogadro constant (6.02 × 10^23^ mol^−1^). This calculation assumes that all stored charges in the system originate from the accumulation of Cl^−^ anions at the interface. The reversible charge/discharge mechanism observed in our case supports this assumption. Figure 5c compares the EPM of Cl^−^ in the NaCl aqueous electrolyte for C-ZnO(NRs) and C-ZnO(NPs) electrodes. It is readily observed that the samples coated with ZnO NRs desalinate more efficiently compared to the samples decorated with only ZnO NPs, in particular at lower current densities. Both samples consume more energy when operating at lower current densities. The EPM traces become stationary at higher current densities, with a minimum of 50 kJ/mol for the C-ZnO(NRs) electrode. Comparing to corresponding experimental data reported in literature in Table 1, our results show a similar energy consumption. Here we mention that publications without a sufficiently substantiated capacitive adsorption mechanism are excluded from this comparison.

**Table 1:** Comparison of energy consumption of different electrode systems.

Electrode material	NaCl concentration (mM)	Potential window (V)	Energy consumption (kJ/mol)	References
Mo_1.33_C-Mxene/CNT	5–600	− 0.8–0.8	53–42	^33^
Porous activated carbon/ion-exchange membranes	10–200	− 1–1.6	55–80	^34^
Active carbon	20	0.6–1	63	^35^
F-doped activated carbon cloth	17	0.8–1.4	153	^20^
Activated carbon	10	0.1–1.2	32	^18^
C-ZnO(NRs)	20	0–0.8	50–100	This work

The electrochemical stability of the electrodes was investigated in experiments where the degradation of the capacitive behavior was monitored versus the number of charge/discharge cycles. Figure 5d shows a less than 10% reduction in the integral areal capacitance of C-ZnO(NRs) electrodes after 500 complete desalination cycles at a current density of 200 μA.cm^−2^.

**Figure 6:**
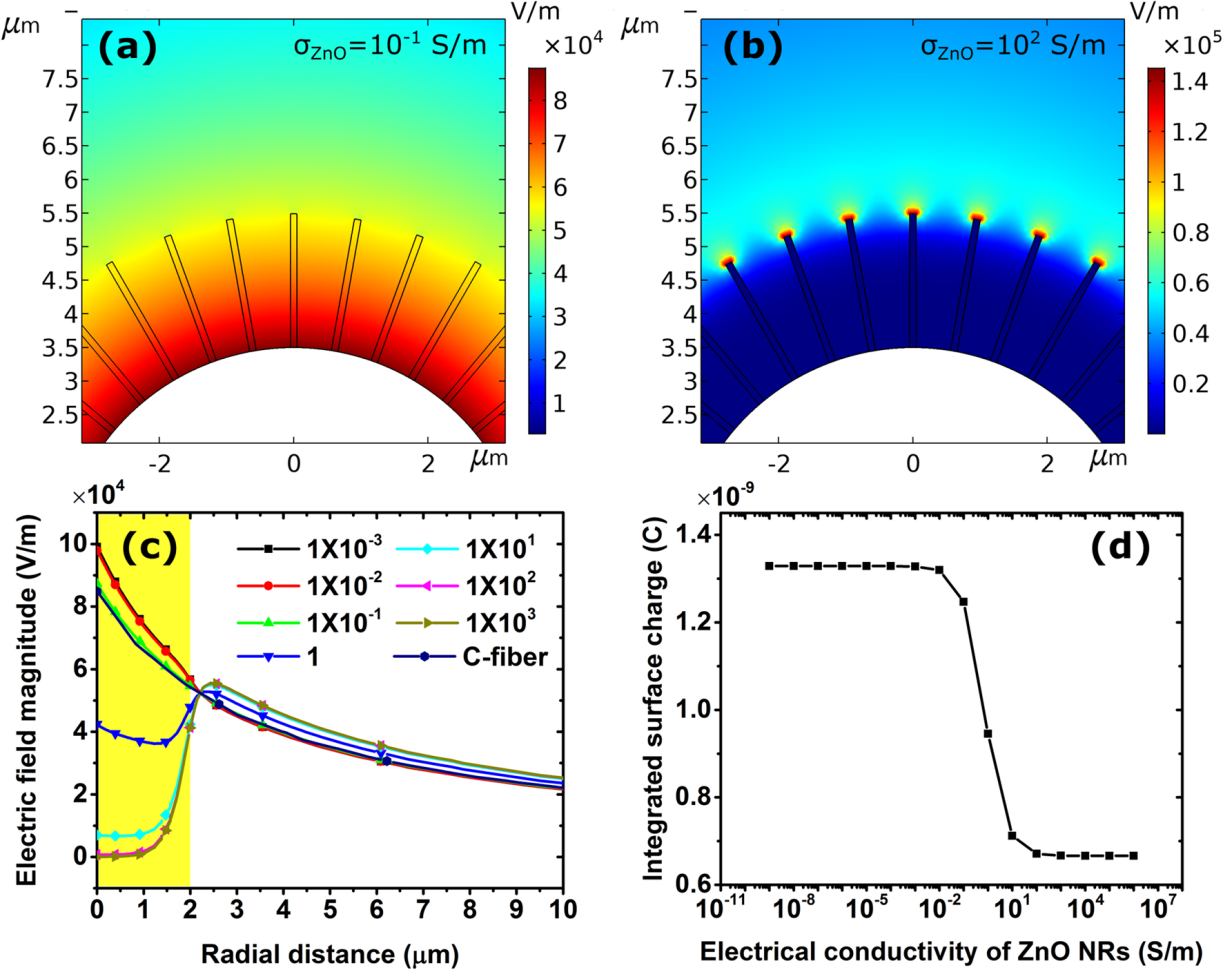
Distribution of the electric field magnitude for a C-fiber (white color) decorated with ZnO NRs with electrical conductivity of (**a**) 10^–1^ S/m and (**b**) 10^2^ S/m. (**c**) Radial profile of the electric field magnitude from the C-fiber surface to a distance of 10 µm for different electrical conductivity (S/m) of the ZnO NRs and for a bare C-fiber without NRs. The yellow area indicates the radial shell that contains the 2 µm long NRs. (**d**) Integrated surface charge on a C-fiber and NRs for different ZnO NR electrical conductivities. The potential of the C-fiber surface is set to 1 V relative to a zero-potential ground at infinity in all simulations.

To clarify the effect of the NRs on the ion adsorption capacity, the electric field distribution for a C-fiber, with and without ZnO NRs, was simulated using COMSOL 5.4. A 2-D finite element method of a 7 µm thick C-fiber, decorated with 2 µm long ZnO NRs with a relative permittivity of 10.4 and varying electrical conductivity in the range of 10^–9^–10^6^ S/m, immersed in a liquid representing brackish water with an electrical conductivity of 0.1 S/m and a relative permittivity of 80 was constructed. The electric field distribution was subsequently calculated by solving Gauss’ law and the continuity equation, using the Electric Currents module with free triangular mesh elements, for different conductivities of the doped ZnO NRs. Figures 6a and 6b show the distributions of the electric field magnitude at an effective electrode potential of 1 V relative to a far point zero reference potential for a C-fiber coated with ZnO NRs with an electrical conductivity of 0.1 and 100 S/m, respectively. The results show that an increased NR conductivity leads to a stronger electric field at the top of the NRs, while shielding the bottom part of the NRs and the C-fiber’s surface. The radial electric field profiles versus NR electrical conductivity in Fig. 6c confirms the shielded electric field in the shell containing the NRs (yellow area). In particular, the electric fields are significantly weaker for higher ZnO NR conductivities while being slightly stronger in the outer region. Considering the fact that the ion adsorption occurs on the ZnO NRs and C-fiber surfaces, an increased conductivity also has the drawback of decreasing the corresponding surface charge (Fig. 6d) and thereby the desalination efficiency of the electrodes. This can explain why the highly Al-doped ZnO NRs did not significantly enhance the electrode capacitance in our experiments (Fig. 4d). Here it should be mentioned that the simulation is based on the stationary study for which the ions in the water medium are immobile. In the real dynamic case, the attracted anions shield the electric field, which leads to more efficient ion adsorption on the NRs and C-fiber surfaces. From these results, it can furthermore be concluded that not only the conductivity of the ZnO NRs influences the final electrode capacitance, but also their length^13^.

The demonstrated commercially available low-cost carbon papers, modified by coating with ZnO nanostructures, show a promising route to develop innovative green electrodes for stable capacitive adsorption/desorption of Cl^−^ anions in brackish water. However, a complete CDI cell requires a complementary second electrode for Na^+^ cation adsorption/desorption. One promising way under development is to combine the electrodes in this work with other suitable materials, e.g., conductive polymers^31^, which can work in the negative voltage range without degradation. Further studies are needed to realize a stable operational asymmetric CDI cell.

## Conclusions

In conclusion, a commercial low-cost carbon paper was homogeneously seeded with a compact ZnO NP layer using a sol–gel technique. Subsequently, ZnO NRs, with and without Al doping, were grown on the seeded carbon paper by a CBD method. The uniform shell of ZnO NRs around each carbon fiber significantly increases the active surface area of the electrodes and enhances their wettability. The CV and CP analyses confirmed a promising capacitive double-layer charge storage mechanism in the electrodes immersed into a brackish electrolyte. The voltage and current dependencies of the areal capacitance of the electrodes were analyzed, and a maximum areal capacitance of 2 mF/cm^2^ was obtained for a C-ZnO(NRs) electrode.

The C-ZnO(NRs) electrodes showed excellent stability after a 500-cycle desalination operation, indicating the regenerative property of the electrodes. These electrodes also exhibited the highest anion adsorption capacity with a minimum energy consumption of 50 kJ per mole of Cl^−^. Simulated electric field magnitude distributions for a C-fiber decorated with ZnO NRs showed a strong dependence of the accumulated surface charge on the conductivity and length of the NRs. To the best of our knowledge, this study is the first demonstration of highly uniform growth and in-depth electrochemical investigations of doped and undoped ZnO NRs on dense networks of C-fibers. Although the electrodes were primarily developed for CDI water desalination, the results can be directly adopted for supercapacitor applications.

## Supplementary Information

Supplementary Information.

## Data Availability

All data relevant for the reproduction of the results presented in this work are included in this publication.
