# Supplementary material for: Electrochemical investigation of carbon paper/ZnO nanocomposite electrodes for capacitive anion capturing
Source: Sci Rep. 2022 Jul 12;12:11843. doi: 10.1038/s41598-022-15771-w (PMC9276741; doi:10.1038/s41598-022-15771-w)
Supplement: Supplementary file 1 — Supplementary Information. [file 41598_2022_15771_MOESM1_ESM.docx]

Supplementary Information

Electrochemical investigation of carbon paper/ZnO nanocomposite electrodes for capacitive anion capturing

Ebrahim Chalangar^1,2^, Emma M. Björk^3^ and Håkan Pettersson^1,2,4*^

^1^ Department of Science and Technology, Physics, Electronics and Mathematics, Linköping University, SE-60174, Norrköping, Sweden

^2^ School of Information Technology, Halmstad University, SE-301 18 Halmstad, Sweden

^3^ Nanostructured Materials, Department of Physics, Chemistry and Biology, Linköping University, SE-581 83 Linköping, Sweden

^4^ Solid State Physics and NanoLund, Lund University, Box 118, SE-221 00 Lund, Sweden

^*^ Correspondence: [hakan.pettersson@hh.se](mailto:hakan.pettersson@hh.se)


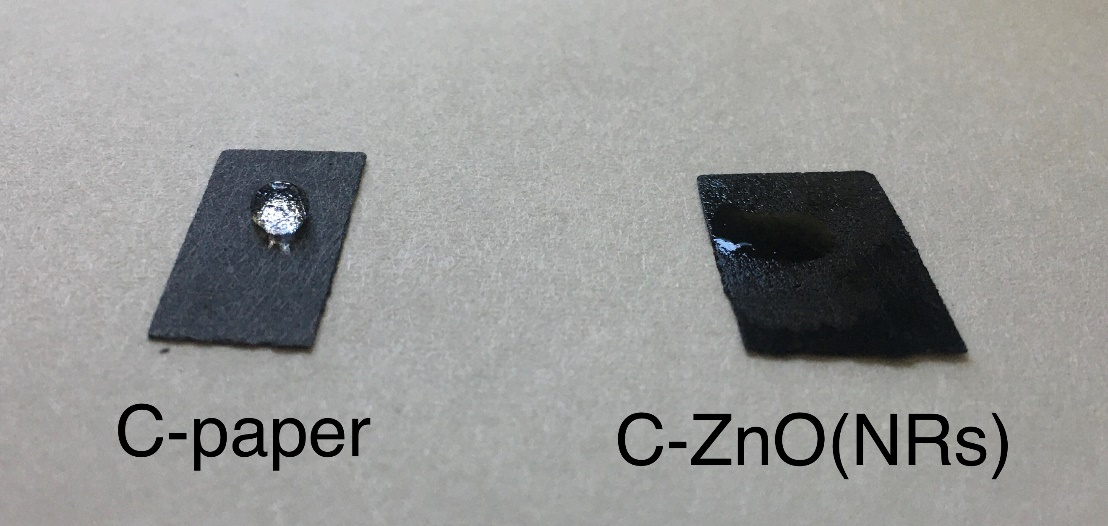


Figure S1. Optical images of a water droplet placed on top of a pure C-paper (left) and on a C-ZnO(NRs) electrode (right), respectively, demonstrating the drastically improved hydrophilicity of the latter.


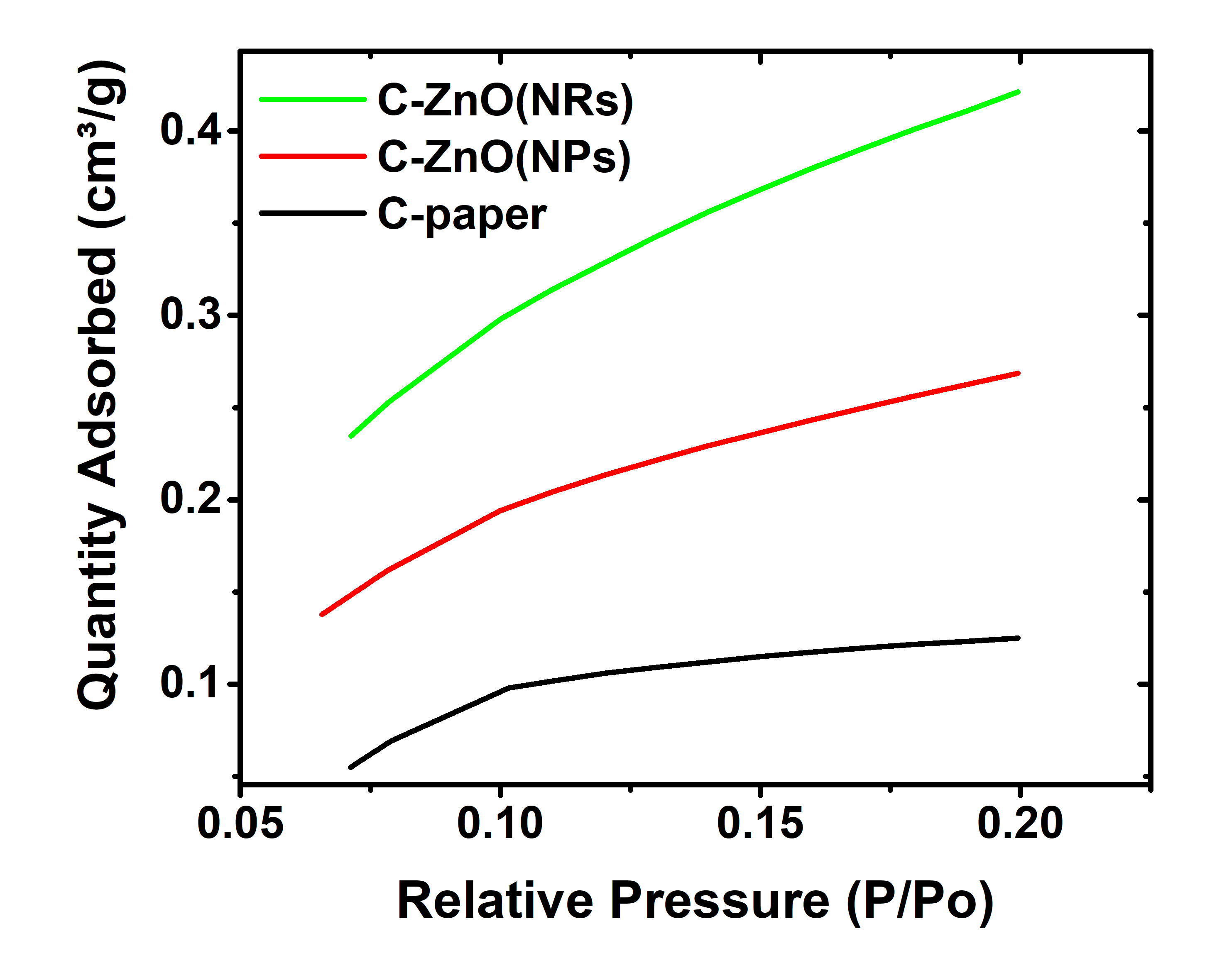


Figure S2. Kr adsorption isotherms for three different electrodes.


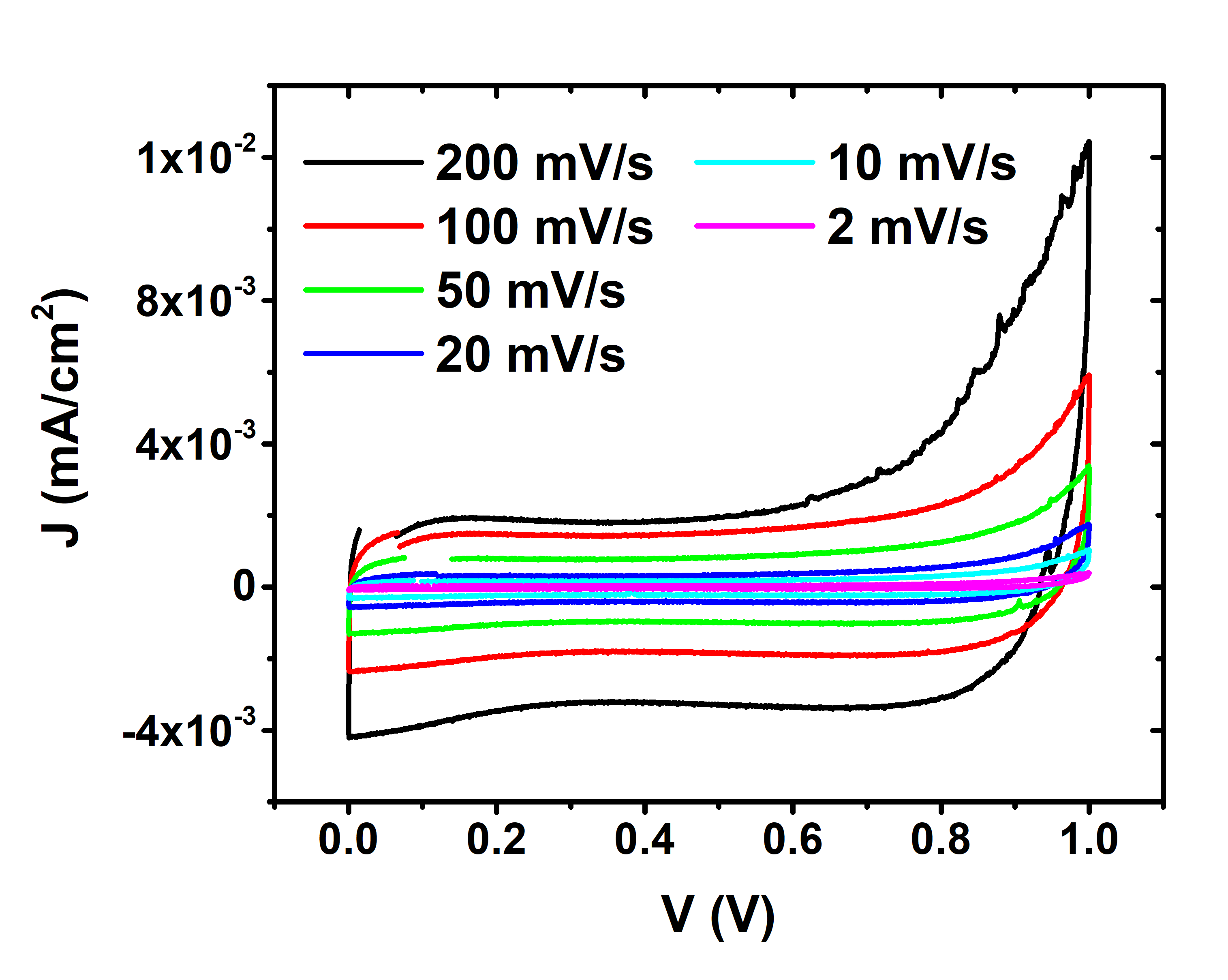


Figure S3. Cyclic voltammograms of a pristine C-paper at different scan rates.


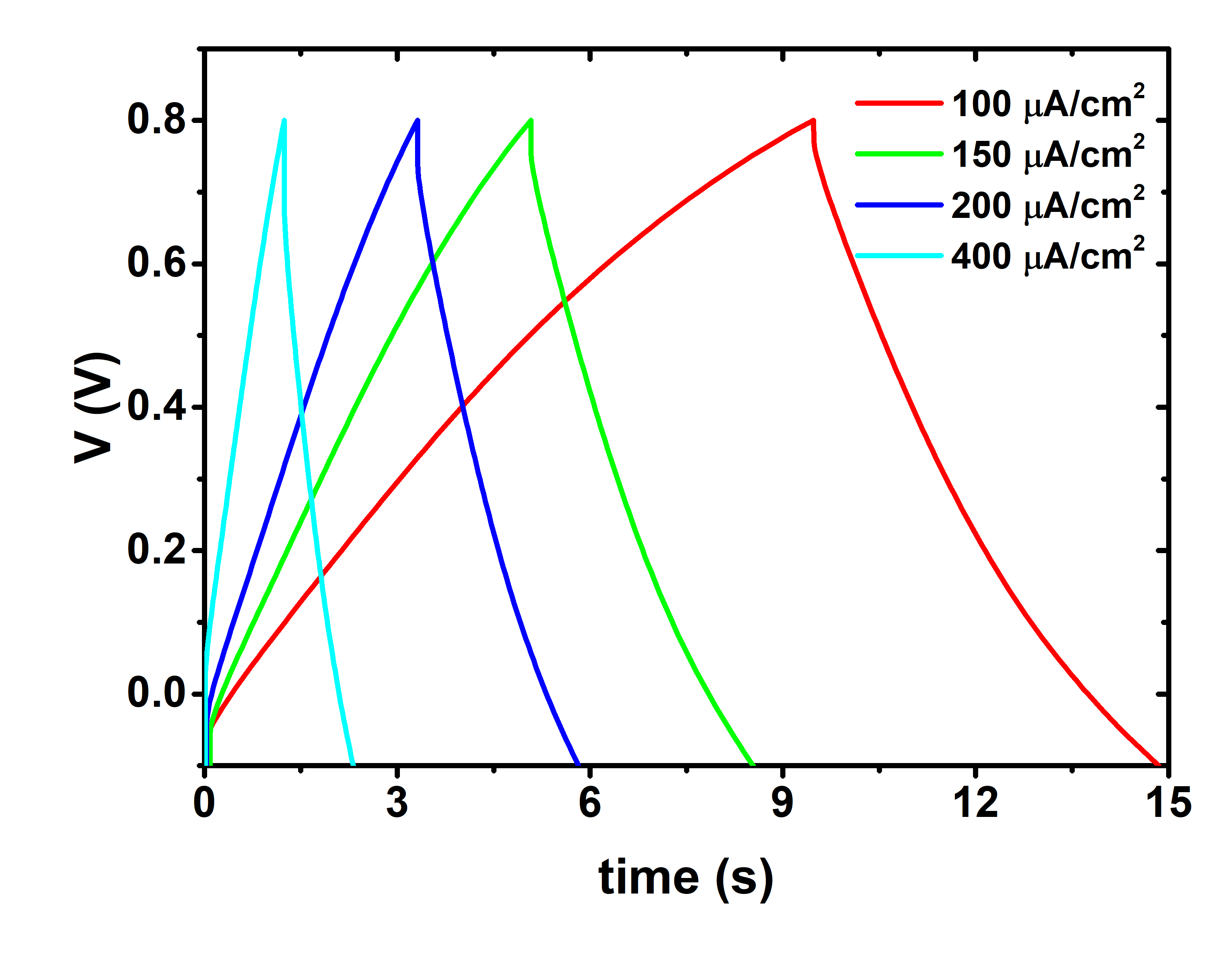


Figure S4. CP curves for a C-ZnO(NPs) electrode at different charging/discharging current densities.
